# Supplementary material for: An α2-Adrenergic Agonist, Brimonidine, Beneficially Affects the TGF-β2-Treated Cellular Properties in an In Vitro Culture Model
Source: Bioengineering (Basel). 2022 Jul 12;9(7):310. doi: 10.3390/bioengineering9070310 (PMC9312232; doi:10.3390/bioengineering9070310)
Supplement: Supplementary file 1 [file bioengineering-09-00310-s001.zip › bioengineering-1790141-supplementary.pdf]

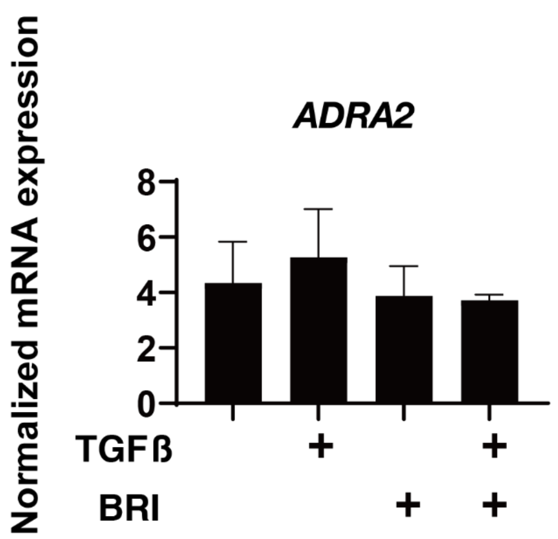

Supplemental Figure S1. The mRNA expression of the α2 adrenergic receptor by HTM cells.

At Day 6, 3D HTM spheroids (control) and those treated with 5 ng/ml TGF-β2 in the absence or presence of 10 μM brimonidine (BRI) were subjected to qPCR analysis to estimate the expression of mRNA in *α2 adrenergic receptor*. All experiments were performed in duplicate using fresh preparations consisting of 16 spheroids each. Data are presented as the arithmetic mean ± the standard error of the mean (SEM).

Supplemental Table S1. Sequences of primers of qPCR.

		Sequence	Exon Location	RefSeq Number	Product Length (bp)
human RPLP0*1	Probe<br>Primer2<br>Primer1	5'-/56-FAM/CCCTGTCTT/ZEN/CCCTGGGCATCAC/3IABkFQ/-3'<br>5'-TCGTCTTTAAACCCTGCGTG-3'<br>5'-TGTCTGCTCCCACAATGAAAC-3'	2-3	NM_001002	143
human COL1A1*1	Probe<br>Primer2<br>Primer1	5'-/56-FAM/TCGAGGGCC/ZEN/AAGACGAAGACATC/3IABkFQ/-3'<br>5'-GACATGTTCAGCTTTGTGGAC-3'<br>5'-TTCTGTACGCAGGTGATTGG-3'	1-2	NM_000088	115
human COL4A1*1	Probe<br>Primer2<br>Primer1	5′-/56-FAM/TCATACAGA/ZEN/CTTGGCAGCGGCT/3IABkFQ/-3′<br>5′-AGAGAGGAGCGAGATGTTCA-3′<br>5′-TGAGTCAGGCTTCATTATGTTCT-3′	51-52	NM_001845	142
human COL6A1*1	Probe<br>Primer2<br>Primer1	5′-/56-FAM/CAGGTTTCG/ZEN/GTCACAGCGGTAGT/3IABkFQ/-3′<br>5'-CCTCGTGGACAAAGTCAAGT-3'<br>5'-GTGAGGCCTTGGATGATCTC-3'	2-3	NM_001848	114
human FN1*1	Probe<br>Primer2<br>Primer1	5′-/56-FAM/TACAGCTTA/ZEN/TTCTCCCTCGCCCAG/3IABkFQ/-3′<br>5'-CGTCCTAAAGACTCCATGATCTG-3'<br>5'-ACCAATCTTGTAGGACTGACC-3'	3-4	NM_212482	129
human αSMA*1	Probe<br>Primer2<br>Primer1	5'-/56-FAM/AGACCCTGT/ZEN/TCCAGCCATCCTTC/3IABkFQ/-3'<br>5'-AGAGTTACGAGTTGCCTGATG-3'<br>5'-CTGTTGTAGGTGGTTTCATGGA-3'	8-9	NM_001613	105
human Grp78*2	Forward<br>Reverse	5'-CATCACGCCGTCCTATGTCG-3'<br>5'-CGTCAAAGACCGTGTTCTCG-3'		NM_005347	215
human GRP94*2	Forward<br>Reverse	5'-CTGGGACTGGGAACTTATGAATG-3'<br>5'-TCCATATTCGTCAAACAGACCAC-3'		NM_003299	152
human XBP*2	Forward<br>Reverse	5'-AGTAGCAGCTCAGACTGCCA-3'<br>5'-CCTGGTTCTCAACTACAAGGC-3'		NM_005080	313
human sXBP*2	Forward<br>Reverse	5'-GGTCTGCTGAGTCCGCAGCAGG-3'<br>5'-GGGCTTGGTATATATGTGG-3'		AB076384	97
human CHOP*2	Forward<br>Reverse	5'-GGAGAACCAGGAAACGGAAAC-3'<br>5'-TCTCCTTCATGCGCTGCTTT-3'		NM_004083	69
Human ADRA2*1	Probe<br>Primer2<br>Primer1	5'-/56-FAM/AGGAAGCGG/ZEN/ATCGTGTGAGGTTTC/3IABkFQ/-3'<br>5'-GCAGTCAGCGTGAGTCTAC-3'<br>5'-TCATCTACACCATCTTCAACCAC -3'	1-1	NM_000681	121

*1 Taqman probes (IDT, Coralville, IA, USA). *2 SYBR probes (IDT, Coralville, IA, USA).
